# Genome-Wide Comprehensive Analysis of the Nitrogen Metabolism Toolbox Reveals Its Evolution and Abiotic Stress Responsiveness in Rice (*Oryza sativa* L.)

**DOI:** 10.3390/ijms24010288

**Published:** 2022-12-24

**Authors:** Zhihui Li, Mingqiang Zhu, Jinqiu Huang, Shan Jiang, Shuang Xu, Zhihong Zhang, Wenchuang He, Wenchao Huang

**Affiliations:** 1 State Key Laboratory of Hybrid Rice, College of Life Sciences, Wuhan University, Wuhan 430072, China; 2 Shenzhen Branch, Guangdong Laboratory of Lingnan Modern Agriculture, Genome Analysis Laboratory of the Ministry of Agriculture and Rural Affairs, Agricultural Genomics Institute at Shenzhen, Chinese Academy of Agricultural Sciences, Shenzhen 518120, China

**Keywords:** nitrogen metabolic genes, phylogenetic analysis, gene expression, co-expression network, qRT-PCR

## Abstract

Nitrogen metabolism (NM) plays an essential role in response to abiotic stresses for plants. Enzyme activities have been extensively studied for nitrogen metabolism-associated pathways, but the knowledge of nitrogen metabolism-associated genes involved in stress response is still limited, especially for rice. In this study, we performed the genome-wide characterization of the genes putatively involved in nitrogen metabolism. A total of 1110 potential genes were obtained to be involved in nitrogen metabolism from eight species (*Arabidopsis thaliana* (L.) Heynh., *Glycine max* (L.) Merr., *Brassica napus* L., *Triticum aestivum* L., *Sorghum bicolor* L., *Zea mays* L., *Oryza sativa* L. and *Amborella trichopoda* Baill.), especially 104 genes in rice. The comparative phylogenetic analysis of the superfamily revealed the complicated divergence of different NM genes. The expression analysis among different tissues in rice indicates the NM genes showed diverse functions in the pathway of nitrogen absorption and assimilation. Distinct expression patterns of NM genes were observed in rice under drought stress, heat stress, and salt stress, indicating that the NM genes play a curial role in response to abiotic stress. Most NM genes showed a down-regulated pattern under heat stress, while complicated expression patterns were observed for different genes under salt stress and drought stress. The function of four representative NM genes (*OsGS2*, *OsGLU*, *OsGDH2*, and *OsAMT1;1*) was further validated by using qRT-PCR analysis to confirm their responses to these abiotic stresses. Based on the predicted transcription factor binding sites (TFBSs), we built a co-expression regulatory network containing transcription factors (TFs) and NM genes, of which the constructed *ERF* and *Dof* genes may act as the core genes to respond to abiotic stresses. This study provides novel sights to the interaction between nitrogen metabolism and the response to abiotic stresses.

## 1. Introduction

Nitrogen (N) is a major element that is widely distributed in plant physiology and metabolic processes. It is used in the synthesis of amino acids, proteins, and secondary metabolites, as well as in signaling several cellular and morphological processes that regulate plant growth and development [1,2,3]. Nitrogen metabolism is a complex dynamic process involving numerous physiological and biochemical processes, including nitrogen uptake, transportation, assimilation, and mobilization [4]. The uptake of N in plants has usually been associated with the inorganic N uptake, i.e., nitrate (NO_3_^−^) and ammonium (NH_4_^+^), from the soil. Prior to nitrogen assimilation, plants’ uptake of nitrate and ammonium is an active process mediated by nitrate transporters (NRT) and ammonium transporters (AMT), respectively. The NO_3_^−^ is firstly transported into the root and then transformed into NH_4_^+^ via nitrate reductase (NR) and nitrite reductase (NiR). After that, NH_4_^+^ is catalyzed by glutamine synthetase (GS) to glutamine (Gln) and further catalyzed as glutamate (Glu) to participate in the amino acid cycle by glutamate synthase (GOGAT) [5]. Previous studies have shown that GDH participates mainly in the deamination of L-Glu, i.e., NH_4_^+^ release [6]. The two main transporter families, including NRT and AMT, are responsible for NO_3_^−^ uptake from the soil. For example, *OsNRT2.3a*, cooperating with *OsNAR2.1*, are responsible for root-to-shoot NO_3_^−^ transport [7], and *OsNRT2.3b* is also involved in transporting NO_3_^−^ to the shoot and remobilizing N into the grain. In plants, the uptake of N (either in nitrate or ammonium form) occurs by specialized transporters present in roots [8]. The reduction of nitrate can take place in the cytoplasm of both root and shoot tissues. Once the nitrate is reduced into nitrite, its further reduction into ammonium takes place in the chloroplast. The final incorporation of ammonium ions into amino acids also takes place in the chloroplast.

Excessive salinity in soils, drought, and high temperatures are the most frequent environmental stress factors that greatly affect plant growth and crop production worldwide [9]. Drought stress is frequently associated with heat stress [10,11]. Under salt stress, enhancing nitrogen (N) assimilation is conducive to plant adaptation to salt stress. For instance, Yang et al. [12] investigated the changes in the activities of NR and NiR in salt-tolerant varieties and sensitive varieties of buckwheat (*Fagopyrum esculentum* Moench.) under salt stress, and the results showed that NR and NiR of the salt-tolerant varieties had a relatively small decrease in activity. Bioinformatic analysis showed that the nitrate transporter *NRT1.8* was up-regulated by multiple stresses, including high salinity, drought, and cold stress, in *Arabidopsis thaliana* (L.) Heynh. [13]. In *Zostera marina* L., NR and NiR genes obtained higher expression levels after the NaCl treatment [14]. Under drought stress, the photosynthetic rate also tends to decrease, depleting the C-skeletons and energy supply. This could lead to a reduction in nitrate reductase (NR) and glutamine synthetase (GS) activities since these enzymes depend directly or indirectly on these substrates. In contrast, a moderate nitrogen supply relieved the drought stress and enhanced the photosynthetic capacity [15,16,17,18]. In cereal crops, such as wheat (*Triticum aestivum* L.) and rice (*Oryza sativa* L.), GS has been thought as an important metabolic indicator of drought tolerance [10,19]. Moreover, GDH has also been recognized as a stress-response enzyme, and it can be induced by elevated ROS levels under abiotic stress [20,21].

Rice is a staple food for nearly half of the world’s population, and its production is urgently needed with the rapid increase of population in coming decades, especially in Asia countries [22]. However, it is estimated that 50% of the world’s rice production is affected by drought [23]. Droughts reduced crop production by 10% from 1964 to 2007 [24]. Projections indicate that 50% of the arable land will be lost by 2050 due to soil salinization [25]. The negative effects of soil water stress on plant N metabolism, such as reduction in N-metabolism enzymes activities and synthesis of nitrogenous compounds, have been widely demonstrated [26]. For example, overexpressing GS genes in rice exhibited resistance to salt, drought, and cold stress [27]. Glutamate dehydrogenase may act as a stress-responsive enzyme in response to abiotic stress. Heterologous expression of a fungal NADP(H)-GDH gene (MgGDH) from *Magnaporthe grisea* (Hebert) Barr can improve dehydration stress tolerance in rice [28]. Although several significant signs of progress have been made in the response mechanism of nitrogen to plant stress at the morphological, physiological and molecular levels, the research on the involvement of nitrogen metabolism in plant stress resistance is relatively scattered. In particular, the systemic and dynamic transcriptional regulation of NM genes’ response to abiotic stress remains fragmented.

In this study, we performed the genome-wide characterization of the genes which were putatively involved in the nitrogen metabolism of land plants from eight representative species, including three dicotyledonous species (*A. thaliana*, *Glycine max* (L.) Merr., *Brassica napus* L.), four monocotyledonous species (*T. aestivum*, *Sorghum bicolor* L., *Zea mays* L., *O. sativa*), and one *Amborella trichopoda* Baill. that was used as an outgroup. Combined with comparative phylogenetic analysis, we identified 104 potential NM genes related to N uptake and assimilation in rice. Through public transcriptome data among different tissues, we initially explored the expression pattern of these NM genes in rice. Then, we investigated the transcriptional changes of these NM genes under three major abiotic stresses, including drought stress, heat stress, and salt stress, via transcriptome data and quantitative real-time PCR (qRT-PCR) experiments. Finally, with the prediction of transcription factor binding sites (TFBSs) on the promoter of these NM genes, we generated a co-expression network consisting of potential transcription factors and NM genes to imply the complicated regulation of NM genes under abiotic stresses. This study can provide a meaningful reference for rice to balance the relationship between nitrogen uptake and stress resistance.

## 2. Results

### 2.1. In Silico Identification of Key Genes Involved in Nitrogen Uptake and Assimilation

According to the genes which were characterized to encode *bona fide* enzymes in nitrogen metabolism through forward or reverse genetic approaches of *A. thaliana*, we conducted a systematic survey of seven gene families of eight representative species which were involved in nitrogen uptake and assimilation to demonstrate the genes that actually encode these enzymes (Figure S1a). These species covered three Dicots (*A. thaliana*, *B. napus*, and *G. max*, four Monocots (*O. sativa*, *S. bicolor*, *T. aestivum*, and *Z. mays*), and 1 Amborellales (*A. trichopoda*) used as an outgroup. The number of genes related to nitrogen metabolism showed diverse density among these species, with significant expansion in the polyploids, i.e., *B. napus* and *T. aestivum* (Figure S1b, Table S1). The NRT1 family was the most abundant family with 716 members in these species, especially containing 219 members in *T. aestivum*. Of note, the genes related to nitrogen metabolism in rice, as a member of the monocot, showed divergence not only with the other monocot species, i.e., *Z. mays* but also with the eudicot species. Such divergence in gene number enabled us to explore the evolutionary relationship between rice and other species.

#### 2.1.1. NR

**Figure 1:**
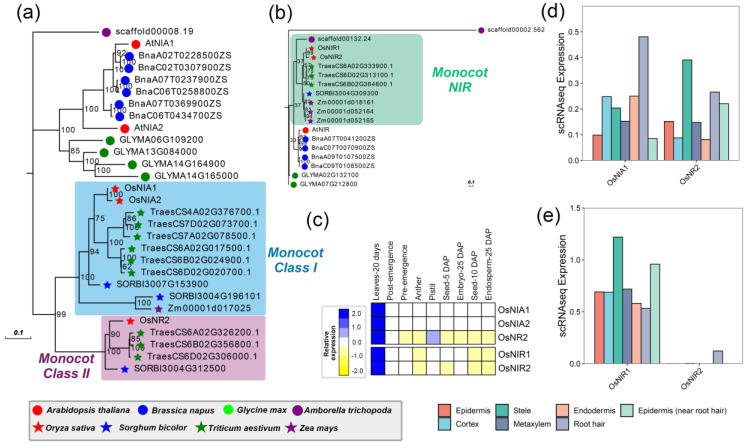
The phylogenetic analysis and expression pattern of NR and NiR superfamily. (**a**,**b**) represent the phylogenetic trees of the NR and NiR superfamily, respectively. The red, blue, and green circle dots represented the three monocot species, including *A. thaliana*, *B. napus*, and *G. max*. The purple circle dots indicated the outgroup *A. trichopoda*. By contrast, the stars of red, blue, green and purple indicated these genes from four monocots, containing *O.sativa*, *S. bicolor*, *Triticum aestivum*, and *Z. mays*, respectively. (**c**) indicates the expression heatmap of NR and NiR superfamily among nine tissues. The tissues names of Nipponbare represent as follows: Leaves-20 days: leaves at 20 days; Post-emergence: emerging inflorescence; Pre-emergence: Early Inflorescence; Anther: anther; Pistil: pistil; Seed-5 DAP: seed five days after pollination; Embryo-25 DAP: embryo 25 days after pollination; Seed-10 DAP: seed 10 days after pollination; Endosperm-25 DAP: endosperm 25 days after pollination. The color bar means that the expression was high with the blue color, while it was expressed at a low level with the yellow bar, and (**d**,**e**) mean the expression of NR and NiR superfamily through the single-cell transcriptome data of roots in rice. The bars with different colors represent the different types of cells of roots.

Nitrate reductase (NR) catalyzes the first step of the general nitrogen assimilation. It catalyzes the nitrate (NO_3_^−^) into nitrite (NO_2_^−^) in the cytoplasm with NAD(P)H. There are two members named *NIA1* and *NIA2*, encoding NR in *A. thaliana*. Ninety percent of the NR activity was conducted by *NIA2*, whereas only the residual 10% of the NR activity was under *NIA1* [29]. Among the eight species, a total of 29 candidate *NR* genes were identified with the most members in *T. aestivum* (9), indicating the individual duplication events in *T. aestivum.* Interestingly, phylogenetic analysis showed that all the members in monocots are clustered together as a sister clade to the clade formed by eudicots members, suggesting that duplication events related to the different paralogs within the *NR* gene occurred after the divergence between eudicots and monocots (Figure 1a). The *NR* members in the monocots can be divided into two classes, named Class I, containing *OsNIA1* and *OsNIA2*, and Class II, which was clustered by the *OsNR2* homologous genes. In Class I, the core genes *OsNIA1* and *OsNIA2* in *rice* (diploid) were clustered with three times the number of *NR* genes in *T. aestivum.* (hexaploid), suggesting the gene duplication events were related to the whole genome duplications during the evolution of *T. aestivum*. However, we observed the distinct gene loss event of *Z. mays* in Class II, implying the significant differentiation on the nitrogen metabolism between the two species. To explore the expression pattern of *OsNR* genes among different tissues, we conducted expression analysis on the transcriptome data of different tissues in *Oryza sativa* cv. Nipponbare (Figure 1c). The results showed that all the *OsNR* genes showed strong and preferential expression in the leaves-20 days, while only *OsNIA1* and *OsNR2* were expressed in the root. Notably, we observed the expression divergence of *OsNIA1* and *OsNR2* in root hair and stele from the single-cell transcriptome data, which could not be distinguished by the traditional transcriptome data (Figure 1d). These results indicated that *OsNRs* might play a diverse role in nitrogen metabolism in the different tissues of plants.

#### 2.1.2. NiR

Nitrite reductase (NiR) is another enzyme that reduces nitrite (NO_2_^−^) with the addition of ammonium (NH_4_^+^). As previously reported, *AtNIR* was involved in nitrate-responsive activity, and mutations within conversed motif in the promoter were proved to reduce the nitrate-responsive activity [30]. A total of 18 *NIR* genes were obtained from the eight species, of which two were from rice. The results of the phylogenetic analysis showed that the *NIR* genes were clustered into two main clades, i.e., eudicot *NIR* and monocot *NIR* (Figure 1b). Monocot *NIR* contained two genes in *O. sativa*, three genes in *T. aestivum*, one gene in *S. bicolor*, three genes in *Z. mays*, and, in particular, one gene in *A. trichopoda*. The results indicated the NIR genes originated from the original ancestor of angiosperms and evolved to have diverse functional differentiation in these species, especially between eudicots and monocots. The two members in rice shared high protein sequence similarities but showed diverse expression patterns. Although these genes were all observed with high expression in Leaves-20 days and low expression in Seed-10 DAP and Endosperm-25 DAP, there was an extreme difference in the different cells of roots, and *OsNIR2* was exactly not detected expressed. The results suggested that *OsNIR*s have undergone functional differentiation during the evolution of plant species.

#### 2.1.3. GS

**Figure 2:**
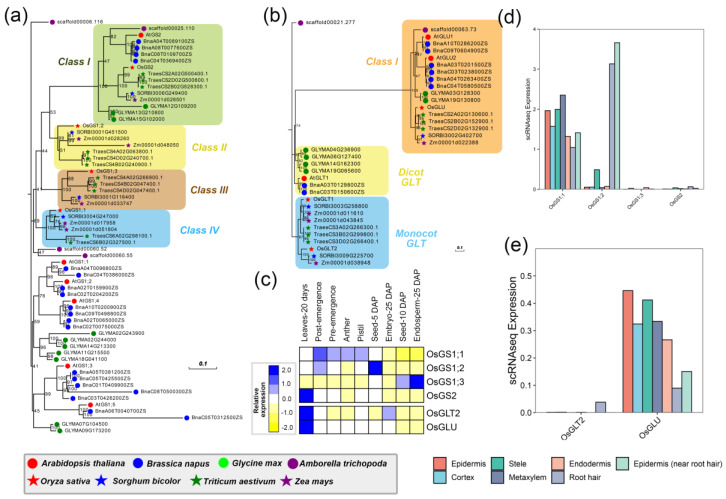
The phylogenetic analysis and expression pattern of GS and GOGAT superfamily. (**a**) and (**b**) represent the phylogenetic trees of the GS and GOGAT superfamily, respectively. The different dots are the same in Figure 2a. (**c**) indicates the expression heatmap of the GS and GOGAT superfamily, and (**d**,**e**) represent the expression of the GS and GOGAT superfamily through the single-cell transcriptome data of roots in rice.

Glutamine synthetase (GS, EC 6.3.1.2) is a key enzyme in the first step of ammonium (NH_4_^+^) assimilation, catalyzing the NH_4_^+^ to the g-carboxyl group of glutamate to produce glutamine and acting as the center for nitrogen flow in plants [31]. There are a total of 64 potential *GS* genes identified among these eight species, of which four potential *GS* genes were identified in rice, named *OsGS1;1*, *OsGS1;2*, *OsGS1;3*, and *OsGS2*. The phylogenetic analysis showed four classes were of importance for monocot NR genes (Figure 2a). The *OsGS2* was clustered with *AtGS2* in Class I, indicating a high sequence similarity between them. However, *GS1* genes showed more divergence among different species of monocots and eudicots. For example, *OsGS1;2* was clustered into monocot Class II with one gene in *S. bicolor* (*SORBI3001G451500*), two genes in *Z. mays* (*Zm00001d028260* and *Zm00001d048050*), three genes in *T. aestivum* (*TraesCS4A02D063800.1*, *TraesCS4D02G240700.1*, and *TraesCS4B02G240900.1*), whereas *OsGS1;3* clustered with other monocots *GS1;3* genes to form Class III and *OsGS1;1* formed Class IV. These results indicated that *OsGS* genes had undergone a dramatic functional divergence, which was further supported by the diverse expression pattern among different tissues. For example, *OsGS1;1* was expressed at a high level in most tissues (Figure 2c), including leaves-20 days, pre-emergence, post-emergence, anther, pistil and seed-5 DAP, indicating that *OsGS1;1* was involved in seed development [32]. Further, the knockout of *OsGS1;1* has a severe impact on growth in rice under normal N conditions [33]. Different from *OsGS1;1*, *OsGS1;2* exhibited high level in seed-5 DAP, where *OsGS1;3* expressed highly in endosperm-25 DAP (Figure 2c). By contrast, *GS2* is usually a single copy in most diploid species and is found in green tissues, such as leaves [34]. It was reported that *AtGS2* was located in mitochondria and chloroplasts of the leaf to participate in ammonium recovery during photorespiration [34]. The conversed function of *OsGS2* could be supported by the extremely high expression in leaves-20 days (Figure 2c). These results suggest a relevant role of *OsGS2* in glutamine biosynthesis associated with photosynthetic metabolism in leaves. Moreover, it was found that *OsGS1;1* and *OsGS2* were highly expressed in different types of root cells by scRNA, especially in root hair and epidermis (near root hair) (Figure 2d). Together results imply a role for these genes in primary assimilation of nitrogen from soil, as it has been previously described for other cytosolic GS enzymes in plants [35,36].

#### 2.1.4. GOGAT

Glutamate synthase (also named glutamine 2-oxoglutarate aminotransferase, GOGAT) converts the transfer of an amide amino group from glutamine to 2-oxoglutarate to form two molecules of glutamate. In plants, GOGAT can be characterized into two types, including ferredoxin-dependent glutamate synthase (Fd-GOGAT, EC 1.4.7.1) and nicotinamide adenine dinucleotide (NADH-GOGAT, EC 1.4.1.14). In *A. thaliana*, three *GOGAT* genes were found in the genome, of which *AtGLT1* was highly expressed in the root to participate in ammonium assimilation [37]. As a result, there were 35 potential *GOGAT* genes that might be involved in ammonium assimilation. Interestingly, as ammonium is the major inorganic nitrogen source for rice, we recovered two members of *NADH-GOGAT*, *OsGLT1* and *OsGLT2*, belonging to the Monocot *GLT* group in rice, which was previously known to facilitate the conversion of soil ammonium to nitrate [38] (Figure 2b). Unfortunately, *OsGLT1* was not detected among these tissues and the root cells. Nevertheless, *OsGLT2* showed modest overall expression among these tissues and was almost exclusively restricted to leaves-20 days. However, the poor expression of *OsGLT2* in different root cells implied a weak function in the root tissue (Figure 2e). Furthermore, it was reported that the *Fd-GOGAT* gene mainly participated in ammonium assimilation derived from photorespiration. Indeed, *OsGLU*, the homologous gene of *Fd-GOGAT* in *O. sativa*, was found to be expressed at extremely high levels in leaves-20 days, while it was expressed at a low level across the cells of the root, including epidermis, stele, metaxylem, and endodermis (Figure 2e). These results indicated that the leaves are mainly the site of *GOGAT* genes in rice.

#### 2.1.5. GDH

**Figure 3:**
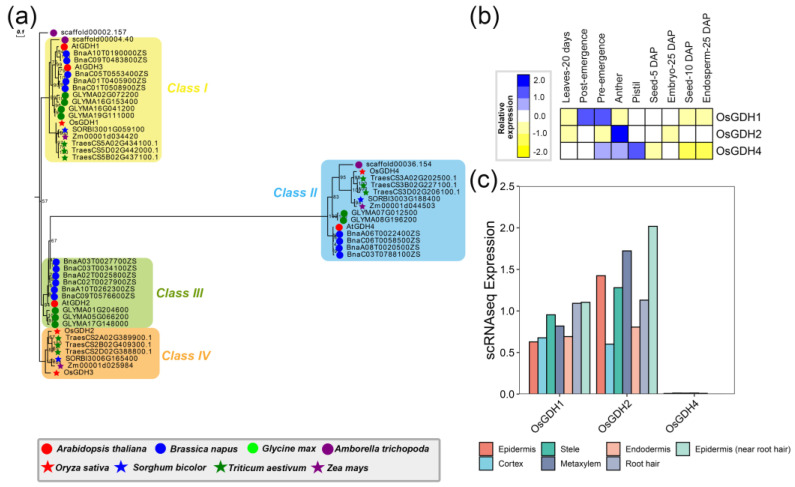
The phylogenetic analysis and expression pattern of the GDH superfamily. (**a**) represents the phylogenetic trees of the GDH superfamily. (**b**) indicates the expression heatmap of the GDH superfamily, and (**c**) represents the expression of the GDH superfamily through the single-cell transcriptome data of roots in rice.

Glutamate dehydrogenase (GDH, EC 1.4.1.2~4) is considered to be a key enzyme in ammonium assimilation. It is a family of enzymes that catalyzes reversible deamination of L-glutamate to 2-oxoglutarate or α-ketoglutarate (α-KG), directly connected to the Krebs cycle [39]. In higher plants, there are two distinct isozymes of GDH [40]. The first one is NAD-specific GDHs which were localized in mitochondria, while the other one was named NADP-specific GDHs exist in chloroplasts [41]. A total number of 50 *GDH* genes were identified among those species, which could be classified into four classes (class I to IV) according to the phylogenetic tree (Figure 3a). The rice genome comprises four members of *GDH*, including *OsGDH1* (from class I), *OsGDH2* (from class III), *OsGDH3* (from class IV), and *OsGDH4* (from class II). The *NADP-GDH* gene (represented as *AtGDH4* and *OsGDH4*) was clustered in class II and showed a higher degree of conservation than *NAD-GDHs*. The widely expressed *OsGDH4* in all tissues revealed that *OsGDH4* is important in the vegetative and reproductive development of *O. sativa* (Figure 3b). However, for *NAD-GDH* genes, class III was only clustered with dicotyledonous genes, indicating that *GDH2* may have diverse functions in nitrogen metabolism between eudicots and monocots. In Class IV, *OsGDH3* was clustered with *OsGDH2* without other genes, which implied *OsGDH3* was independently duplicated by *OsGDH2*. Moreover, the expression results showed that *OsGDH1* was expressed very highly and preferentially in pre-emergence and post-emergence, while *OsGDH2* was expressed preferentially in anther (Figure 3b). As expected, *OsGDH3* was detected with a low expression or undetected across the cells of the root, suggesting that *OsGDH3* had a significant functional divergence. *OsGDH4* was modestly expressed in most tissues (Figure 3b). The scRNA showed that *OsGDH1* and *OsGDH2* were expressed across most of the root cells, especially with a high level in root hair and epidermis (near root hair) (Figure 3b). Together the results suggested that *OsGDH* genes may have diverse functions during the development of rice.

#### 2.1.6. AMT

**Figure 4:**
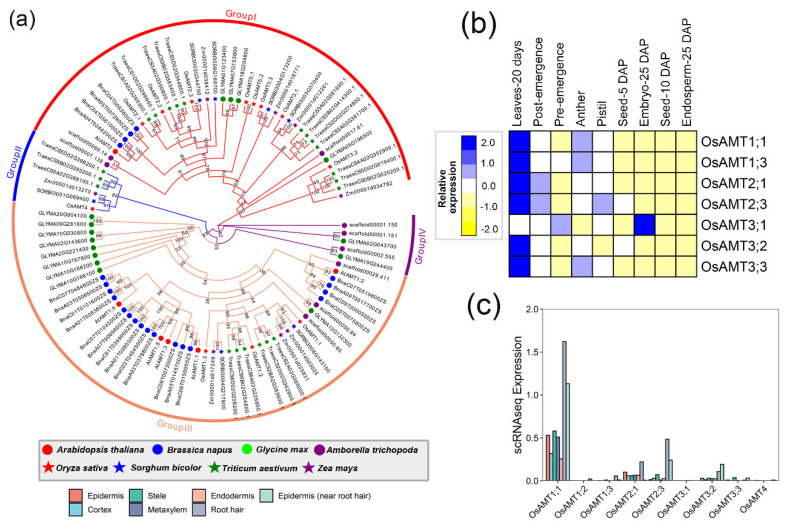
The phylogenetic analysis and expression pattern of AMT superfamily. (**a**) represents the phylogenetic trees of the GDH superfamily. The genes from the same cluster were defined as the same group. Based on the branch, the whole members were classified into four groups. (**b**) indicates the expression heatmap of the AMT superfamily, and (**c**) represents the expression of the AMT superfamily through the single-cell transcriptome data of roots in rice.

Ammonium transporters (AMTs) are the key proteins that transport ammonium/ammonia from extracellular into intracellular locations. The assimilation from NO_3_^−^ to NH_4_^+^ costs 12–26% of photosynthesis energy, and the use of NH_4_^+^ as an N source conserves a large amount of energy for plants [42]. In plants, ammonium is taken into root cells by AMTs and then synthesized by glutamine synthase (GS) [43]. As a result, a total of 97 potential *AMT* genes were retrieved and then classified into four groups (Figure 4a). A subset of seven genes in *O. sativa*, including *OsAMT2;1*, *OsAMT2;2*, *OsAMT2;3*, *OsAMT3;1*, *OsAMT3;2*, *OsAMT3;3*, *OsAMT3;1*, *OsAMT5;1*, and *OsAMT5;2*, were clustered in Group I with *AtAMT2*, indicating the *AMT2* genes were independently duplicated after the divergence between monocots and eudicots. Group II was a monocots-specific clade with *OsAMT4* and five other monocots genes, implying the potential role of *AMT4* in monocots. Within Group III, *AMT1* genes from monocots form a clade nested among *AMT1* genes from eudicots., indicating a single origin of these genes. To further investigate the functional divergence of these genes, we analyzed the expression pattern of *OsAMT* genes. As a result, most of the *OsAMT*s exhibited weak expression among these nine tissues, except for leaves-20 days, revealing the curial role of *OsAMT*s in the development of the leaf (Figure 4b). Likewise, the expression of *OsAMT*s in scRNA pointed to the potential role of *OsAMT1;1* in the different root cells (Figure 4c).

#### 2.1.7. NRT

**Figure 5:**
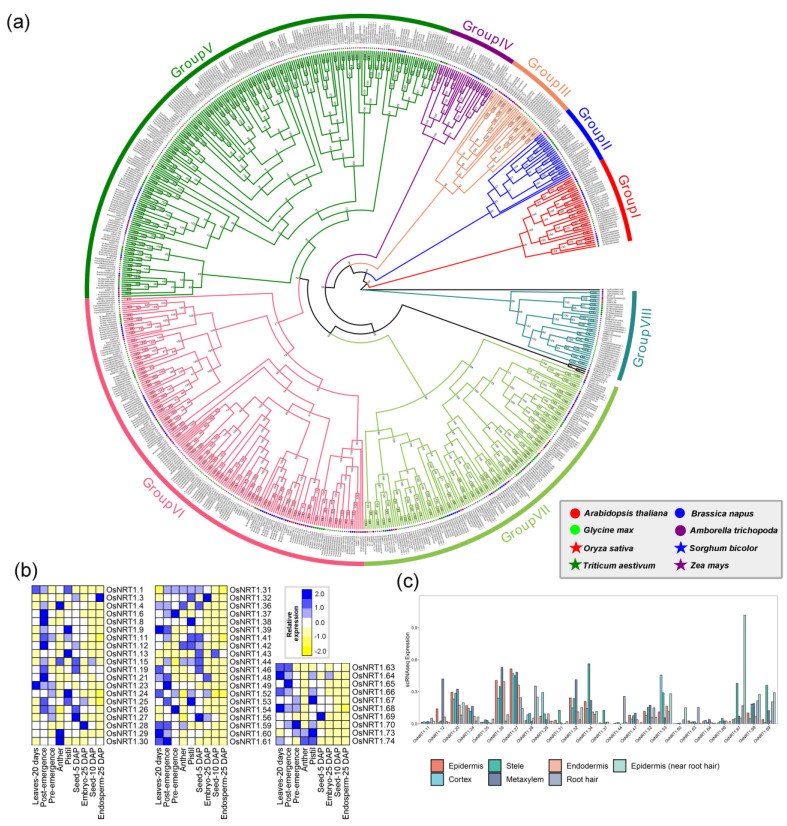
The phylogenetic analysis and expression pattern of the NRT1 superfamily. (**a**) represents the phylogenetic trees of the NRT1 superfamily, splitting into seven groups. The different colors on the branches were defined as the same group. (**b**) indicates the expression heatmap of the NRT1 superfamily, and (**c**) represents the expression of the NRT1 superfamily through the single-cell transcriptome data of roots in rice.

**Figure 6:**
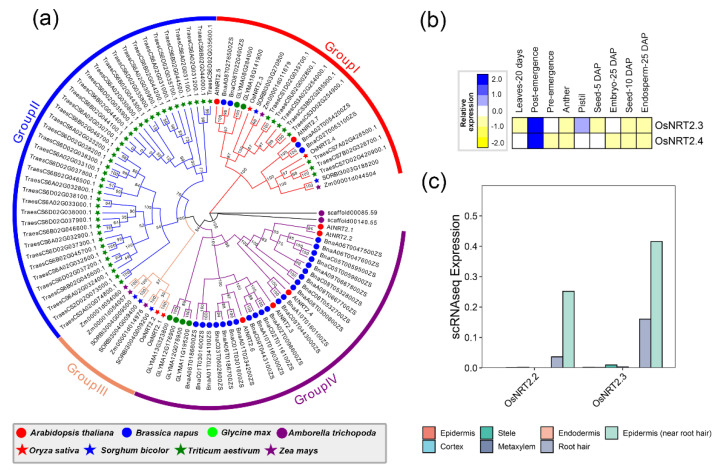
The phylogenetic analysis and expression pattern of the NRT2 superfamily. (**a**) represents the phylogenetic trees of the NRT2 superfamily, splitting into four groups. The different colors on the branches were defined as the same group. (**b**) indicates the expression heatmap of the NRT1 superfamily. *OsNRT2.1* and *OsNRT2.2* were filtered with lower expression, and (**c**) represents the expression of NRT2 superfamily through the single-cell transcriptome data of roots in rice.

A large proportion of the NO_3_^−^ acquired by plants from soil is actively transported through NO_3_^−^ transporters (NRT) [44]. In higher plants, there are two types of nitrate transporters, known as *NRT1*s and *NRT2*s. *NRT2*s are high-affinity nitrate transporters, while most *NRT1*s are low-affinity nitrate transporters; *NRT2* activity supplants *NRT1* activity as N availability diminishes [45]. The plant nitrate transporters are a large family in that we excavated a total of 716 potential *NRT1* genes and *101* potential *NRT2* genes. Among them, 74 *OsNRT1* genes and four *OsNRT2* genes were observed in *O. sativa* (Figure 5 and 6). The *NRT1* gene family could be divided into eight groups, named Group I-VIII, with extensive duplication and multiplication. The expression pattern of *OsNRT1s* showed that some members expressed only in one tissue while others displayed a broad expression profiling. For example, *OsNRT1.27*, *OsNRT1.28*, *OsNRT1.42*, *OsNRT1.44*, and *OsNRT1.53* exhibited high expression among these seven tissues (Figure 5b). *OsNRT1.1* of Group VII, which has a major role in nitrogen metabolism, showed high expression in post-emergence, leaves-20 days, anther and pistil. *OsNRT1.43*, belonging to Group V, had the highest expression level in seed-5 DAP, seed-10 DAP and endosperm-25 DAP. *OsNRT1.68, OsNRT1.64, OsNRT1.52, OsNRT1.48, OsNRT1.13, OsNRT1.26* and *OsNRT1.11* showed modest expression among these nine tissues (Figure 5b). The expression of scRNA showed *OsNRT1.67* was specifically expressed in the epidermis (near root hair), while *OsNRT1.12*, *OsNRT1.26*, and *OsNRT1.32* were dominant in metaxylem and *OsNRT1.34* in stele (Figure 5c).

The *NRT2* gene family was divided into four groups, named groups I–IV (Figure 6a). *OsNRT2.3* and *OsNRT2.4* were distributed into group I, while *OsNRT2.1* and *OsNRT2.2* were clustered in group III. The uneven difference of *OsNRT2*s implies that these *NRT2*s probably originated from different ancestors and underwent a functional divergence. As expected, *OsNRT2.1* and *OsNRT2.2* were not detected in the issues, while *OsNRT2.3* and *OsNRT2.4* showed dominant expression in post-emergence (Figure 6b). However, *OsNRT2.3* and *OsNRT2.4* exhibited similar expression profiles in the epidermis (near root hair) of root cells, indicating that *OsNRT2.2* may share a similar function in the root (Figure 6c).

### 2.2. Expression Profiling of Genes Related to Nitrogen Metabolism Using Transcriptome Data under Different Abiotic Stresses

**Figure 7:**
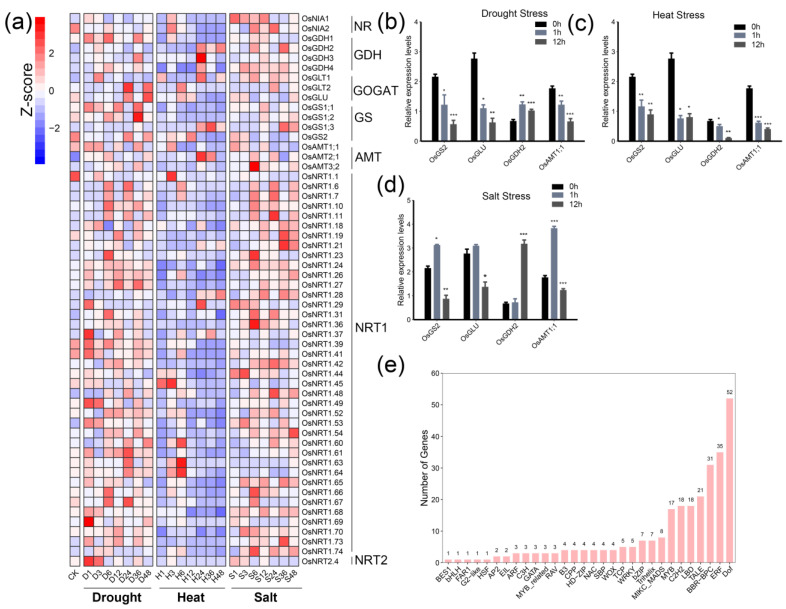
The diversification of NM genes under abiotic stresses. (**a**) shows the expression of all NM genes under three abiotic stresses, including drought stress, heat stress, and salt stress. The genes with average FPKM < 1 were filtered. (**b**), (**c**) and (**d**) indicate the relative expression of four genes, including *OsGS2*, *OsGLU*, *OsGDH2*, and *OsAMT1;1*, by qRT-PCR validation. The bar “0 h” means the control. The bar “1 h” means 1 h after treatment, while “12 h” means 12 h after treatment, and (**e**) indicates transcription factors binding site on the promoter (2000 bp before the transcript start sites) predicted by PlantTFDB. “*” indicates *p* < 0.05, “**” indicates *p* < 0.01, while “***” indicates *p* < 0.001.

To investigate the potential mechanism of nitrogen metabolism under abiotic stresses, we collected the transcriptome data of rice under three major abiotic stresses, including drought stress (DS), heat stress (HS), and salt stress (SS), to characterize their transcriptional profiling. As a result, we observed a significant expression divergence among the members belonging to the same family (Figure 7a). For instance, in the first enzyme NR family, two members, including *OsNIA1* and *OsNIA2*, were identified with transcriptional changes under abiotic stress. At the same time, the *OsNR2* was filtered out because of the low expression (mean FPKM < 1) or even unexpressed. It was found that the expression of *OsNIA1* rapidly increased in 1 h and reached a stable level in control under DS and SS, while *OsNIA2* was the opposite trend with a down-regulated expression under these stresses. The second enzyme was the NiR family, whose members were expressed with a lower level or even not expressed. For the GDH superfamily, four members exhibited an almost strong up-regulated pattern under DS and SS. For example, four members of *OsGDH*s were significantly up-regulated from 1 h to 6 h under DS and SS, especially *OsGDH1*. By contrast, the down-regulation of *OsGDH*s under HS implied the diverse role of *OsGDH*s’ response to abiotic stresses. Two of the *GS* members, including *OsGLT1* and *OsGLT2*, were up-regulated under salt stress, while the other member *OsGLU* was down-regulated under HS, SS and DS in the short term. The *OsGS1*s were up-regulated under DS and down-regulated under HS, while *OsGS2* was up-regulated from 24 to 48 h under HS and from 36 to 48 h under SS. The gene *OsAMT1;1* from the AMT superfamily was rapidly up-regulated from 1 h to 3 h and then decreased under DS, demonstrating that *OsAMT1;1* has a crucial function in the early response to stress. Notably, both *OsAMT2;1* and *OsAMT3;2* showed an increasing expression pattern throughout the whole time, implying its important role in response to stress. In the *NRT1* family, most genes showed up-regulation in DS and SS and down-regulation in HS. However, there were several genes up-regulated at the early stage under HS. For instance, *OsNRT1.63* and *OsNRT1.64* were highly expressed with the FPKM values (6.33 and 4.37 in 3 h, 16.3 and 6.77 in six h), which was more than two times than the control (2.92 and 2.53). In the *NRT2* family, *OsNRT2.4* rapidly increased the expression level from 1 h to 3 h under DS and SS, while it was down-regulated under HS stress.

To further check the accuracy of the above results, we randomly selected four genes, including *OsGS2*, *OsGLU*, *OsGDH2*, and *OsAMT1;1*, for validation of the expression response to abiotic stresses by qRT-PCR. It was found that *OsGS2*, *OsGLU*, and *OsAMT1;1* showed a gradually decreased expression of a significant level under DS, while *OsGDH2* was up-regulated at 1 h (*p* < 0.05) and 12 h (*p* < 0.001) (Figure 7b). *OsGS2*, *OsGLU*, *OsGDH2*, and *OsAMT1;1* exhibited down-regulated expression under HS, implying its potential role in response to HS (Figure 7c). Interestingly, we observed a complicated pattern of these genes under SS (Figure 7d). For example, *OsGDH2* was significantly (*p* < 0.05) up-regulated at 1 h and then significantly (*p* < 0.01) down-regulated at 12 h under SS, indicating that *OsGS2* was involved in response to salt at the early stage. Likewise, *OsGLU* and *OsAMT1;1* shared a similar expression pattern with *OsGS2* at 1 h and 12 h under SS. However, *OsGDH2* was up-regulated after salt stress treatment both at 1 h and 12 h compared with the control, thus suggesting that *OsGDH2* may have a different mechanism to respond to stresses from the other three genes. These results implied the curial role of NM genes within diverse pathways under abiotic stress.

### 2.3. Diverse Potential Regulators of Nitrogen Metabolism Genes Response to Abiotic Stress

It was known that transcription factors (TFs) were involved in multiple biological processes, especially stress response. To further instigate the potential regulators of the above 56 NM genes related to stress, we performed transcription factors binding sites (TFBSs) analysis based on the 2000 bp sequence upstream of genes. A total of 350 TFBSs were predicted among the 56 NM genes, which were classified into 30 TF families (Figure 7e, Table S2). The most abundant type was ERF TFBS, with 142 sites, followed by Dof (58 sites), BBR-BPC (27 sites), and LDB (18 sites). In the NR family, both *OsNIA1* and *OsNIA2* might be regulated by bZIP families, with an additional CPP binding site in *OsNIA2*. In the *GDH* family, it seems to be complicated in the composition of TFBS. *OsGDH1* contained one TFBS of the MIKC_MADS family, while *OsGDH2* had three Dof sites, two BBR-BPC sites, two MYB sites, and one C3H site. *OsGDH3* might be regulated by ARF and CPP TF with one ARF site and one CPP site. Moreover, *OsGDH4* shared one BBR-BPC site with *OsGDH2* with two additional EIL sites. The results suggested that four members of *GDH* genes may be involved in the diverse pathway. *OsGLT1* and *OsGLU*, two members of the GOGAT family had a high similarity on ERF sites and TALE sites, while *OsGLT1* shared one LBD site and one TCP site with *OsGLT2*. In the GS family, *OsGS1;1* contained one BBR-BPC site, one ARF site, one B3 site, one NAC site, and one bHLH site, indicating that *OsGS1;1* may be involved in a diverse biological process by cooperating with different TF genes. The homologous gene *OsGS1;3* might be regulated by Dof family and BBR-BPC family. In the AMT family, there were few sites were identified, including two Dof sites in *OsAMT2;1* and *OsAMT3;2*, two BBR-BPC sites in *OsAMT1;1* and *OsAMT3;2*, two MYB sites in *OsAMT2;1*, and one Trihelix site in *OsAMT3;2*. Moreover, most genes of the NRT1 family were regulated by ERF families, Dof families, and BBR-BPC families. For example, *OsNRT1.1*, *OsNRT1.10*, and *OsNRT1.26* contained 22, 18, and 19 sites of ERF families, respectively, suggesting that they were more likely to be regulated by ERF transcription factors. There were three, three, one and three sites of Dof families in *OsNRT1.11*, *OsNRT1.18*, *OsNRT1.19*, and *OsNRT1.23*, respectively, indicating that they might be involved in the regulatory network of Dof transcription factors. For the NRT2 family, *OsNRT2.4* contained six sites of two different TF families, including two Dof TFBSs and four TCP TFBSs. These results indicated that the nitrogen metabolism-related genes might be targeted by many TF genes to participate in diverse biological processes, especially in response to abiotic stress.

**Figure 8:**
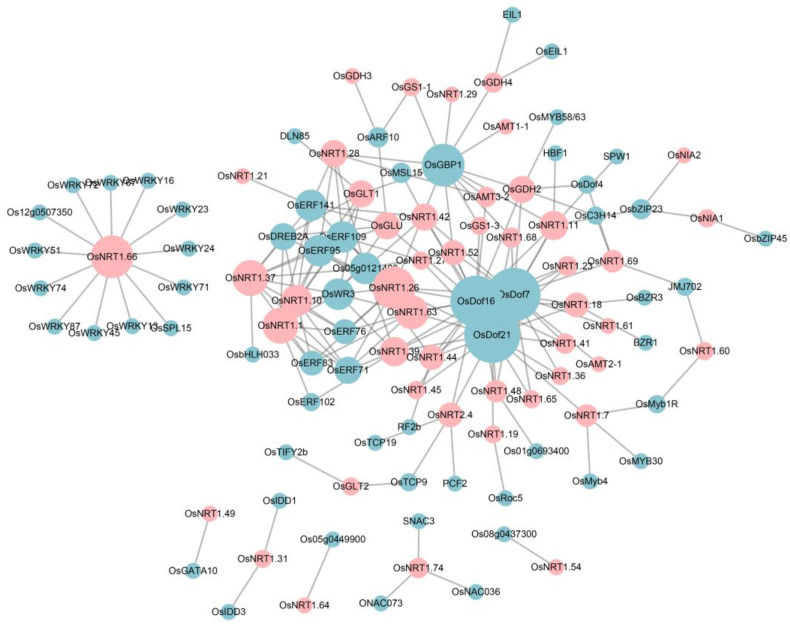
The co-expression regulatory network constructed by the NM gene and transcription factors (TFs). The cyan circles indicate the potential TFs which may regulate the NM genes. The pink circles indicate the NM genes which were involved in response to abiotic stress. The size of the circles represents the degree of the genes in the network.

To understand the potential mechanism of NM genes with TF genes, we construct a co-expression regulatory network based on the expression of TF genes and NM genes under abiotic stress. As above TFBS, we observed the significantly clustered Dof and ERF TF genes in the core of the network, including *OsDof21*, *OsDof7*, *OsDof16*, *OsWR3*, *OsERF141* and *OsERF109* (Figure 8). For instance, *OsDof21* was predicted to have a positive correlation with 19 genes, containing 18 *NRT1* genes and one *NRT2* gene. *OsDof16* was highly positively correlated with *OsGS1;3* and *OsAMT3;2*, while it was negative with *OsGLU*. There were a few genes with specific TF genes which did not overlap with the others. For example, *OsNRT1.49* was specifically regulated by *OsGATA10*. *OsNRT1.74* was simultaneously regulated by multiple NAC transcription factors, such as *SNAC3*, *OsNAC036*, and *OsNAC073*. The most abundant gene was *OsNRT1.66*, which was negatively targeted by 13 WRKY genes, i.e., *OsWRKY16*, *OsSPL15*and *OsWRKY72*. Overall, these results suggested that those genes associated with nitrogen metabolism incorporated with TFs to respond to abiotic stress.

## 3. Discussion

In plants, Nitrogen (N) is a primary plant nutrient that plays a crucial role in determining plant growth and productivity, while several processes, including N uptake and assimilation, are known to be adversely affected by abiotic stresses [46]. However, in the case of rice, the effect of abiotic stress on nitrogen metabolism has been studied only at the enzymatic level so far. Here, we used a comprehensive genome-wide analysis of the phylogenetic relationships and expression profiles of the nitrogen metabolism gene families in rice, highlighting those members that are likely to be involved in nitrogen metabolism and analyzing the expression patterns of these genes in response to abiotic stresses.

The environmental stresses and selection pressures accelerate the evolution of elaborate mechanisms in plants to cope with stresses. Given the highly variable environmental conditions, one of the effective ways of increasing evolution novelties was gene duplication [47]. In this study, we identified three *NR*, two *NiR*, four *GS*, three *GOGAT*, four *GDH*, 10 *AMT*, 74 *NRT1*, and four *NRT2* genes in rice, respectively. A comparative analysis of the phylogenetic relationships among the genes was performed. The results revealed a great deal about the diversification and conservation of the nitrogen metabolism gene family in plants. The conservative phylogenetic relationship was observed in *NR* and *NIR*, indicating the paralogs of *NR* and *NiR* may be originated from a single origin from their respective ancestors after the divergence between monocots and eudicots. Numerous gene duplications were observed in most NM gene families, especially the NRT1 subfamily, which has the most abundant members in those families. Additionally, two homologous genes (*OsAMT1;2* and *OsAMT1;3*) in the AMT superfamily were hypothesized to be the result of gene duplication. However, the expression pattern of those genes under abiotic stress treatment showed a quietly different tendency, indicating the neofunctionalization of duplicated NM genes in rice has gained more power to respond to environmental stresses during its evolution. Overall, gene duplication is a powerful way for plants to adapt to different environments during their development and growth [48].

Tissue-specific expression pattern analysis provided valuable clues about the important roles involved in NM genes in rice growth, development and stress response. Previous reports showed that NR activity under salinity stress was reduced in several plants, like barley, maize and tomato [49,50,51]. On the contrary, increased expression of the *NR2* gene was reported in the roots of *A. thaliana* during the first week when treated with 100 mM NaCl [52]. In rice, salt-responsive cultivars (IR64-sensitive) showed a decrease in NR activity under in vitro and in vivo conditions, whereas CSR 36-tolerant showed an increase in NR activity [53]. Our study of *NR* genes (*OsNIA1* and *OsNIA2*) showed an increasing trend under salt stress, especially *OsNIA1* (Figure 7a). Compared with *NR*, the function of *NiR* under abiotic stresses remained a fragment. In rice seedlings and creeping bentgrass, water stress significantly increased nitrite reductase (NiR) activities [54,55]. Interestingly, NiR was suppressed under heat stress and provoked under salinity in *Citrus aurantium* L. [56]. Under NaCl and/or PEG treatments, there was a significant decrease in NiR activity in bean leaves [57]. Likewise, the NiR in this study was not detected with expression or low expression, indicating that rice plants regulate NO-generating sources when submitted to environmental stress. GS is responsible for ammonium assimilation by catalyzing the conversion of glutamate and ammonium into glutamine [58,59]. Our results showed that the expression of *OsGS2* declined when exposed to three stresses. Therefore, the reduction in the expression of the glutamine synthetase gene under most of the stress might be due to the low availability of glutamate. Reduced expression of the *GS* gene may slow down the process of ammonia assimilation and thus affects nitrogen metabolism. However, *OsGS1;1* was upregulated when exposed to drought treatments and downregulated under heat stress. The reciprocal expression trend of *GS2* and *GS1* genes indicated the *GS* genes had undergone functional differentiation during the evolution of the species. In higher plants, ammonium (NH_4_^+^) assimilation occurs mainly through the glutamine synthetase/glutamate synthase (GS/GOGAT) pathway [60]. GOGAT is a rate-limiting enzyme in this pathway. Under water stress, NH_4_^+^, which was generated through NR and NiR reduction of NO_3_^−^, was reduced as well as the activities of GS and glutamate synthase (GOGAT). Thus, it led to ammonium accumulation and toxic effects on plants [61]. In the present study, *OsGLU* was observed to be downregulated under drought, heat and salt stress, which was similar to the result in *Malus prunifolia* [62]. Moreover, in *ipomoea batatas*, drought stress reduced all the activities of N-metabolism enzymes and the transcriptional levels of nitrate reductase (NR), glutamine synthetase (GS) and glutamate synthase (GOGAT) [63]. The metabolic changes triggered by salt stress result in a decrease in both activities and protein abundance of the key enzyme, namely GOGAT, in barley [64]. It has been shown that *GDH* expression can be induced by elevated ROS levels under abiotic stress [65]. In *Brassica juncea* L., the activity of NADPH-GDH and NADH-GDH showed a significant increase under drought treatment [66]. Likewise, drought stress induced an increase in GDH activity in leaves of *S. alterniflora* [21]. In this study, the increased *OsGDH1* expression was observed under drought stress. These results indicated that *OsGDH1* was induced under drought stress, thereby improving dehydration stress tolerance in plants. Under salt stress, *OsGDH2* was up-regulated. It has been reported that salt stress clearly elevated the expression levels of *OsGDH2* and *OsGDH3* in old leaves, while strongly down-regulated the expression of *OsGS2* and *OsFd-GOGAT* in old leaves [67]. We speculate that excesses of Na^+^ and Cl^−^ even changed the pathway of NH_4_^+^ assimilation in old leaves, then weakening GOGAT/GS pathway and elevating the GDH pathway. Generally, ammonium (AMTs) and nitrate transporters (NARs and NRTs) mediate NH_4_^+^ and NO_3_^−^ transport, respectively. Thus, their expression level may reflect the absorption and subsequent utilization of each N source [68,69]. Under abiotic stress, plants can control the expression level of AMTs, thereby affecting the perception and assimilation of NH_4_^+^. For example, *SlAMT1-2* and *SlMAT1-1* were downregulated under drought stress in *Solanum lycopersicum* L. [70]. It has been reported that the application of NH_4_^+^ can effectively alleviate the adverse effects of drought on the growth and development of rice [71]. In this study, *OsAMT2;1* was up-regulated in response to drought stress, which was consistent with the results in *Populus simonii* carr. and *Malus hupehensis* (Pamp.) Rehd [72,73]. The first step of NO_3_^−^ assimilation is its uptake with the help of nitrate transporters located on the plasma membrane of the root epidermal and cortical cells. In higher plants, two types of nitrate transporters have been found, low affinity nitrate transporters (NRT1) and high affinity nitrate transporters (NRT2). It has been reported that some nitrate transporters were involved in response to drought stress in most plants. For example, the levels of the *MdNRT2.4* transcript from plants under water deficit were upregulated in apples [74]. In sweet potato seedlings, the transcriptional level of nitrate transporter genes *NRT1.1* in leaves and roots was upregulated under drought stress [63]. In rice, compared with wild-type (WT), the survival rate was significantly improved in *OsNAR2.1* over-expression lines and decreased in *OsNAR2.1* RNAi lines after drought stress conditions and irrigation [75]. In our results, *OsNRT1.1* was downregulated, and *OsNRT2.4* was up-regulated from 1h to 3h and down-regulated from 6 to 48 h under drought stress. These observations suggested that the downregulation of two important nitrate transporter genes, *OsNRT1.1* and *OsNRT2.4*, under drought stress conditions disrupts both low- and high-affinity nitrate transport systems, which may be one of the key factors, inhibiting the growth and development of *O. sativa*.

The interaction network of nitrogen metabolism proteins with TF families was also predicted based on the relation-based method [76]. It is found that rice nitrogen uptake and assimilation proteins interacted with the ERF, Dof and BBR-BPC TF family. AP2/ERF family transcription factors are known to regulate diverse processes of environmental stress responses in higher plants, such as abiotic stresses (cold, heat, drought, salinity and osmotic stress) [77,78,79]. Overexpression of *OsAP37* (*OsERF3*) in rice resulted in tolerance to drought during the vegetative and productive stages [80]. *OsERF19*-overexpression plants displayed enhanced tolerance to salt stress and ABA hypersensitivity compared to wild-type and control plants [81]. Apart from this, the Dof family was related to the response to abiotic stress in rice. For example, *OsDof15*, which regulates primary root elongation, is repressed by salt stress [82]. The Overexpression of *OsDof1* in rice can significantly increase the seed-setting rates under cold stress [83]. Overall, these results provide novel sights with potential regulators for nitrogen metabolism under abiotic stresses.

## 4. Material and Methods

### 4.1. In Silico Identification of Nitrogen Metabolism Pathway Genes

The whole-genome protein sequences of eight representative species were downloaded from Ensemble Plants (http://plants.ensembl.org/index.html). In order to identify the N metabolism-related genes, a combination of keywords and BLASTp searches (proteins from *A. thaliana* were used as queries) was conducted [84]. Firstly, we performed an extensive literature survey related to nitrogen metabolism genes in *A. thaliana*. The corresponding protein sequences of genes previously characterized as the nitrogen metabolism toolbox of *A. thaliana* were used as queries in hmmsearch to detect the conserved Hidden Markov Model (HMM) domain [85]. For example, the conserved domain of Glutamine synthetase (GS) was Gln-synt_C (PF00120), and the preliminary GSs in other species were filtered with the domain of Gln-synt_C. Secondly, all the preliminary genes were used as queries in BLASTp with a threshold of e-value < 1 × 10^−5^ to detect the homologs in *A. thaliana*. For nitrogen metabolic genes in *O. sativa*, the whole protein in the rice genome was conducted a detection of HMM domain to filter the candidate nitrogen metabolic genes with the corresponding domain for per superfamily. Next, the above genes were aligned against the nitrogen metabolism toolbox of *A. thaliana* with a threshold of e-value < 1 × 10^−5^ using the BLASTp software. The proteins under the above criteria were used for subsequent analysis. The nitrogen metabolic genes in the other six species were identified with the same pipeline. The genes of *A. thaliana* and *O. sativa* were renamed as the corresponding name in previously reported literature.

### 4.2. Phylogenetic Analyses

For each superfamily, the phylogenetic relationships among those species were analyzed respectively. Firstly, the software MAFFT was used to align the protein sequences of selected sets of nitrogen metabolism [86]. Secondly, substitution models were automatically selected using the ModelFinder, and the maximum-likelihood phylogenetic trees were constructed by IQ-TREE using ultrafast bootstrap approximation (1000 replicates) for branch support [87]. The trees were classified and visualized by the online program Evolview [88]. The different species were represented by different colors and shapes.

### 4.3. RNA-Seq and Single-Cell Transcriptome Expression Analysis

Raw RNA-seq data of rice from PRJNA530826 was retrieved from the NCBI SRA database. It was generated from shoots tissues of rice at seven time points post heat, drought and salt stress at 0 h, 1 h, 3 h, 6 h, 12 h, 24 h, 36 h, and 48 h, respectively. The expression data of different tissues were downloaded from Rice Genome Annotation Project (RGAP) (http://rice.uga.edu/expression.shtml, accessed on 1 July 2021). Single-cell transcriptome data (with the ID GSE146034) of rice root was obtained from Gene Expression Omnibus (GEO). In order to obtain data suitable for cluster displays, the absolute number of fragments per kilobase of transcript per million mapped reads (FPKM) was used in further expression pattern analysis. The genes with an average FPKM < 1 were removed. The heatmap plot was visualized by the “pheatmap” package in R.

### 4.4. qRT-PCR Expression Analysis

The rice variety Nipponbare was grown in Yoshida solution in a greenhouse at 28 °C under a 14 h day/10 h night cycle. Two-week-old seedlings were subjected to heat, drought, and salt stresses following the methods of Byun [89]. For the heat stress treatment, seedlings were incubated at 45 °C [46]. For the drought stress treatment, rice seedlings were placed into a 20% polyethylene glycol 4000 (PEG-4000) solution. For the salt stress, the seedlings were irrigated with 200 mM NaCl solution. Total RNA was extracted from shoot tissues collected at 0, 1, and 12 h after the onset of the abiotic stress imposition. Total RNA was extracted with TRIzol (Invitrogen, Carlsbad, CA, USA) reagent. According to the manufacturer, RNA sample (~ 2 μg) was treated with DNaseI and then used for cDNA synthesis with an ABScript III RT Master Mix with gDNA Remover (ABclonal, Wuhan, China). Oligonucleotide primers for ubiquitin gene (*LOC_Os03g13170*) were used as the internal control for establishing equal amounts of cDNA in all reactions. Primers used are listed in Supplementary Table S3.

### 4.5. Prediction of Transcription Factors Binding Sites (TFBSs) and Construction of Co-Expression of NM Genes

In order to investigate the potential mechanism of NM genes under abiotic stresses, only the genes detected by the transcriptome data were retained for further prediction. The promoter sequences (−2000 bp sequence before the transcript start sites (TSSs)) of NM genes were extracted and then used as a query for prediction of the potential TFs regulation information by the PlantTFDB database with a threshold *p*-value < 10^−6^ (http://planttfdb.gaolab.org/, accessed on 24 October 2016). To obtain the potential regulation between TF genes and NM genes, only the TF genes which contained the binding sites on NM genes of the above TFBSs results were retained for further analysis. After the filtration, 105 potential TF genes were kept. Then, we calculated the Pearson correlation coefficient (PCC) of the whole gene set with the *p*-value < 0.05. The regulatory network was generated by the software Cytoscape v3.8.2 [90].

## 5. Conclusions

We conducted a systematic analysis of 104 genes associated with Nitrogen metabolism in rice. A comparative analysis of the phylogenetic relationships among the genes was performed. The results revealed a great deal about the diversification and conservation of the nitrogen metabolism gene family in plants. Numerous gene duplications were observed in most families, especially the NRT1 subfamily, which has the most abundant members in those families. Additionally, two homologous genes (*OsAMT1;2* and *OsAMT1;3*) in the AMT superfamily were hypothesized to be the result of gene duplication. Moreover, expression patterns from RNA-Seq, single-cell transcriptome data and qRT-PCR analysis demonstrated that genes involved in Nitrogen metabolism showed spatial and temporal regulation in tissue (or stress)-specific way, indicating they played important roles in plants’ growth, development and stress responses.

## Data Availability

The data will be available from the corresponding author upon reasonable request.

## Supplementary Materials

The following supporting information can be downloaded at: https://www.mdpi.com/article/10.3390/ijms24010288/s1.
