# Supplementary material for: Genome-Wide Comprehensive Analysis of the Nitrogen Metabolism Toolbox Reveals Its Evolution and Abiotic Stress Responsiveness in Rice (Oryza sativa L.)
Source: Int J Mol Sci. 2022 Dec 24;24(1):288. doi: 10.3390/ijms24010288 (PMC9820731; doi:10.3390/ijms24010288)
Supplement: Supplementary file 1 [file ijms-24-00288-s001.zip › Figure S1.pdf]

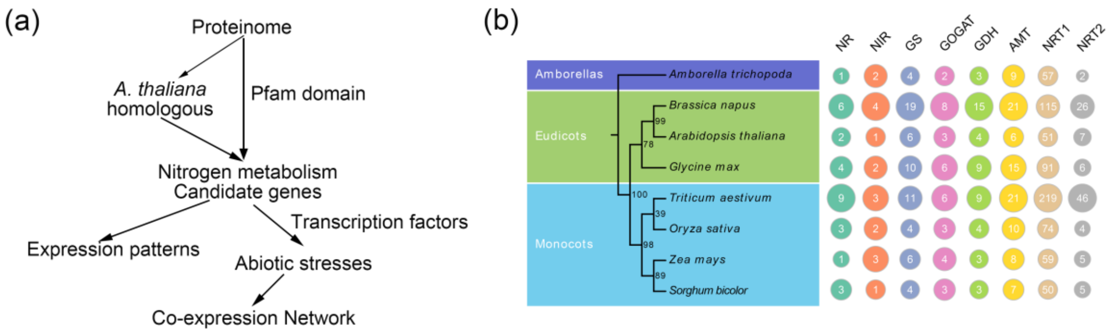

Figure S1. The overview of identification of nitrogen metabolism genes in 8 representative species. (a) The workflow of genome-wide identification on nitrogen metabolisms for per super family. Firstly, we collected the whole genome protein sequence from public dataset (detail see Table S1). Secondly, we used software hmmsearch to detect the conserved Hidden Markov Model (HMM) domain for the whole genome protein sequences. Combining with the homologous identify with the nitrogen metabolism genes in *A. thaliana*, the genes sharing similarity with the genes *A. thaliana* were considered as the potential NM genes. Further, we investigate the phylogenetic relationship and expression pattern of NM genes in rice, and dissect the response to abiotic stresses, thus constructing the co-expression network. **(b)** The overview of NM genes identified in this study. The 8 species was classified into three main types, including Eudicots, Monocots, and Amborellas which was used as the outgroup. The size of dots indicated the number of members in every super family.
